# 3D Medical Collaboration Technology to Enhance Emergency Healthcare 

**Published:** 2009-04-19

**Authors:** Greg Welch, Diane H Sonnenwald, Henry Fuchs, Bruce Cairns, Ketan Mayer-Patel, Hanna M. Söderholm, Ruigang Yang, Andrei State, Herman Towles, Adrian Ilie, Manoj Ampalam, Srinivas Krishnan, Vincent Noel, Michael Noland, James E. Manning

**Affiliations:** 1 Department of Computer Science, University of North Carolina at Chapel Hill, Campus Box 3175, Chapel Hill, North Carolina 27599-3175; 2 Swedish School of Library and Information Science, University of Gothenburg and the University of Borås, SE-50190 Borås, Sweden; 3 Department of Surgery, University of North Carolina at Chapel Hill, Campus Box 7228, Chapel Hill, North Carolina 27599-7228; 4 Department of Computer Science, University of Kentucky, 232 Hardymon Building, Lexington, Kentucky 40506-0195

## Abstract

Two-dimensional (2D) videoconferencing has been explored widely in the past 15–20 years to support collaboration in healthcare. Two issues that arise in most evaluations of 2D videoconferencing in telemedicine are the difficulty obtaining optimal camera views and poor depth perception. To address these problems, we are exploring the use of a small array of cameras to reconstruct dynamic three-dimensional (3D) views of a remote environment and of events taking place within. The 3D views could be sent across wired or wireless networks to remote healthcare professionals equipped with fixed displays or with mobile devices such as personal digital assistants (PDAs). The remote professionals’ viewpoints could be specified manually or automatically (continuously) via user head or PDA tracking, giving the remote viewers head-slaved or hand-slaved virtual cameras for monoscopic or stereoscopic viewing of the dynamic reconstructions. We call this idea remote 3D medical collaboration. In this article we motivate and explain the vision for 3D medical collaboration technology; we describe the relevant computer vision, computer graphics, display, and networking research; we present a proof-of-concept prototype system; and we present evaluation results supporting the general hypothesis that 3D remote medical collaboration technology could offer benefits over conventional 2D videoconferencing in emergency healthcare.

## INTRODUCTION

In [41] we presented some early methods and results for a multi-year interdisciplinary research project to develop and evaluate technology for 3D telepresence, designed to support medical collaboration across geographic distances. We refer to this concept as three-dimensional medical collaboration (3DMC) technology. Here we report collectively on more final results of the project, including advances in the fundamental technologies, the construction of a functioning proof-of-concept 3DMC prototype, and some preliminary results from a multi-year human subject evaluation.

**Figure 1:**
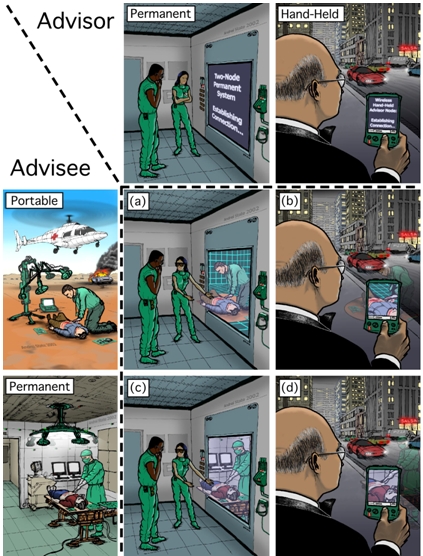
Future vision of 3D telepresence technology for medical collaboration. The left column illustrates examples of person-portable and permanent 3D telepresence technologies used by an advisee. The top row illustrates examples of head-tracked and hand-held technologies used by an advisor. Images (a)-(d) illustrate the shared sense of presence for corresponding advisor-advisee scenarios.

Our long-term 3DMC vision is to enhance and expand diagnosis and treatment capabilities in life-critical trauma situations. We aim to connect two entities: (1) a medical advisee and patient, such as a paramedic treating a trauma victim in the field, with (2) a healthcare specialist acting as medical advisor, for example an emergency room physician at a large medical center. Our goal is to provide a high-fidelity visual and aural sense of presence, such that advisee and advisor can more effectively communicate and share information when diagnosing and treating a patient (see Figure 1). Primarily, but not exclusively, we envision this technology enabling better patient care through extemporaneous medical collaboration across geographic distances in dynamic situations where patient diagnosis and treatment are time-critical and complex, but where physical co-presence of medical experts and patients is not possible.

The basic technical idea for 3DMC is to use a relatively small number of cameras to “extract” (estimate) a time-varying three-dimensional (3D) computer model of the advisee’s remote environment and of the events taking place within. When coupled with head (or hand-held viewer) position and orientation tracking, this should offer the advising physician a continuum of user-controlled dynamic views of the remote scene, with both direct and indirect depth cues through binocular stereoscopy and (in the case of head tracking) head-motion parallax. Figure 1  illustrates a number of example scenarios. We hope that in the future such view-dependent 3D telepresence could be a standard part of mobile emergency patient care systems (e.g., [1]) that today use radio, cell phone or state-of-the-art 2D videoconferencing technology. 

We hypothesize that the shared sense of presence offered by view-dependent 3D telepresence will be superior to current 2D videoconferencing, improving collaboration between geographically-separated medical personnel, enabling new opportunities to share medical expertise throughout, between, and even beyond medical facilities. To investigate this hypothesis, our research addresses two fundamental questions: can we develop the technology for 3D telepresence in medicine, and will the technology be useful to the medical community? Consequently, our research consists of three inter-related components: 3D technology research, prototype development, and evaluation. 

Our project explores the key technological and social barriers to 3DMC today. Technological barriers include real-time acquisition and generation of user-specified views, tracking at the advisor’s site, displays that deliver accurate 3D depth cues and motion parallax, and network congestion and variability issues. We built a prototype system that permits investigation of these technological barriers. 

Social barriers include understanding the potential utility of 3DMC in healthcare and obstacles to its adoption. If the proposed technology will not improve emergency healthcare, or if it will not be adopted and used within the medical community, there is little purpose to continue the technical research. We therefore conducted a posttest, a controlled between-subjects experiment simulating an emergency healthcare situation to examine the potential utility of 3DMC compared to 2D videoconferencing and paramedics working alone. To examine 3DMC’s adoption potential, we conducted interviews with a variety of stakeholders in the medical community, aiming to identify challenges faced by and opportunities resulting from 3DMC adoption and use.

Three different disciplines, computer science, information science and medicine, have been vital to exploring technological and social barriers to 3DMC. Computer scientists have provided visualization and telecommunications technology expertise; information scientists have provided socio-technical evaluation and social informatics expertise; and physicians have provided medical and emergency healthcare expertise. Without each of these disciplines, the project would not have been possible. This is often the case in use-inspired research [2] that examines issues arising in basic research and practice, and strives to contribute to basic research and practice as our project does. This paper synthesizes the disciplinary work done in the project, presenting all project components in a holistic manner for the first time. 

## PREVIOUS RESEARCH

### Medical Collaboration via Videoconferencing Technology 

Medical collaboration, specifically collaboration between different types of healthcare providers and patients, using two-dimensional (2D) videoconferencing and televideo technology, has been explored in a variety of medical settings, such home-based healthcare [3, 4], prison-based healthcare [5, 6] and rural healthcare [7, 8]. Two limitations with respect to the technology are repeatedly emphasized in the literature: the difficulty associated with obtaining the desired camera views, and limitations in depth perception. 

For example, camera view difficulties are mentioned multiple times in the final report for the U.S. National Library of Medicine’s National Laboratory for the Study of Rural Telemedicine [7]. One example is in the discussion of the use of a 2D televideo system to observe children with swallowing disorders. The report states: “Limitations of telemedicine services for management of feeding and growth issues include the need to rely on the interpretations of others during physical exam. At times the camera angles were not ideal to allow for clear pictures of the mouth during feeding” [7, p. 110].

The problem was also identified by Ellis and colleagues [6] where they describe work using a computer-based telemedicine system for semi- and non-urgent complaints at a short-term correctional facility. “The lack of remote control on the patient care camera at the remote site by the examining emergency medical physicians requires the nurse to spend considerable time operating the camera and responding to technical instructions…it was another important reason for nonuse” [6, p. 92].

Patients have also found this same limitation in 2D video technology. Georgetown University Medical Center [9] reports that in contrast to a face-to-face visit, the use of 2D video technology limits the physician’s view of the patient, and as a result patients felt that the physician could not always “see” how the patient was “really doing.”

One could try and address the visibility problem using multiple cameras. But switching between numerous disjoint views, as a security guard might with a surveillance system, is generally not intuitive; nor is it usually feasible in time-critical healthcare situations. With a very large number of cameras and tracking of the user’s head motion, one could imagine automatic switching based on view position and orientation. But the quantity and configuration of cameras necessary to achieve near-seamless and appropriate switching over an operating room, as well as the associated 2D video storage and bandwidth needs, would be impractical. While pan-tilt-zoom cameras can help address this problem, they require additional technical skills, impose an additional cognitive load onto the advisor, and require additional time to adjust (which is difficult in a trauma situation). 

In addition to the challenges in obtaining the desired 2D view of a remote patient, Tachakra states “impaired depth perception is a significant problem in telemedicine.” and notes that “the most important cue of depth is due to binocular disparity” a [10, p.77]. Similarly, a university “Clinical Studio” which used video conferencing to perform neurological examinations reported: “[Video-conferencing technology is not difficult and can be [handled]… by [Emergency Room]… staff. However the images are in two dimensions hence certain aspects of the exam could be enhanced by more than one camera angle.”[7, p. 187] 

In situations where depth perception would aid in the collaboration, advisee medical personnel today must resort to secondary visual cues or to verbal clarification from a remote advisor. Both impose additional cognitive loads compared to the very natural views afforded if the advising physician were able to “be there” with the patient. Tachakra describes several “coping strategies” that could be used to overcome the inherent limitation of the 2D imagery. Chief among the coping strategies is the practice of “rotating the camera in the transverse plane about 30° at a time” [10, p. 83]. Tachakra surmises that this controlled camera rotation “enables the consultant to build a three-dimensional mental image of the object by briefly storing a range of two-dimensional views” [10, p. 83]. This is not surprising given that object occlusion and motion parallax: b are two of the most powerful depth cues. 

However, it is often not realistic to require camera rotation as prescribed by Tachakra in emergency, time-critical healthcare situations in the field. For example, the time needed to rotate a camera and view the rotation reduces the amount of time available to perform life-saving procedures. It reduces the number of on-site personnel who can provide assistance to a trauma victim, as it requires the full-time effort of a trained on-site person. Moreover, in some situations it may be physically very difficult to rotate a camera, e.g., when a victim of a car accident is lying on an incline along the side of a road. To address these limitations, we are developing 3D telepresence technology that inherently provides depth perception and dynamic views. 

### Sense of Presence and Task Performance via 3D Technology

Previous research shows that in general, 3D technology enables an increased sense of presence, i.e. subjective perception of being present or immersed within a synthetic, usually computer-generated environment, often referred to as “Virtual World” or “Virtual Environment” (VE). For example, Hendrix and Barfield [11] report on three studies where they vary display parameters and attempt to assess a user’s sense of presence. The results from the first and second study indicate that the reported level of presence is significantly higher when head tracking and stereoscopy cues are provided. The third study indicates that the level of presence within the VE increases with the visual field of view.

There is also evidence to suggest that view-dependent or immersive 3D displays increase users’ task performance. For example, in a study of how various system parameters affect the illusion of presence in a VE, Snow [12] reports a moderately positive relationship between perceived presence and task performance. Pausch and colleagues [13] found that users performing a generic pattern search task perform roughly twice as fast when they change from a stationary 2D display to a head-mounted (and tracked) 3D display with identical properties. Schroeder and colleagues [14] present the results of a study in which distant collaborators attempted to solve a Rubik’s cube type puzzle together. The authors compare face-to-face task performance with networked performance using both an immersive 3D display and a conventional 2D desktop display. They report that task performance using the networked immersive 3D display and in the face-to-face scenario were very similar, whereas desktop performance was “much poorer.” Most recently, Mizell and colleagues [15] describe a 46-person user study aimed at determining whether immersive 3D virtual reality demonstrates a measurable advantage over more conventional 2D display methods when viewing and interpreting complex 3D geometry. The authors report that the head-tracked 3D system shows a statistically significant advantage over a joystick-controlled 2D display.

Thus previous research suggests that a 3DMC system may potentially improve information sharing and task performance in emergency medical situations, possibly leading to improve patient care. Yet there are significant technical challenges that must be overcome in order to create a 3DMC system.

### 3DMC Technical Challenges 

To create a 3DMC system, we must reconstruct a dynamic remote 3D scene in real time. The most common approach to 3D scene reconstruction is to use multiple cameras and effectively “triangulate” points in the observed scene. This involves automatically picking some visual feature in one camera’s 2D image, finding the same feature in a second camera, and then mathematically extending lines from those cameras into the scene. The place where the lines intersect corresponds to the 3D location of the feature in the room. If one can do this reliably for a sufficient number of points in the scene, many times per second, then with some assumptions about the scene, and with a lot of compute power, one can turn the dynamic collection of disjoint 3D points into a coherent dynamic (that is, time-varying) 3D computer model that one can use like a flight simulator.

However, there are at least three areas of fundamental difficulty associated with trying to reconstruct dynamic 3D models of real scenes: feature visibility, feature quality, and reconstruction algorithms. Features might not exist or might be confusing or ambiguous. They are often hard to detect, isolate, resolve, and correlate, and automating the overall reconstruction process in light of these difficulties is a very hard problem. The state of the art is limited to static environments for large spaces, or dynamic events in relatively small, well-controlled environments.

To address these challenges, our multi-year project sponsored by the U.S. National Institutes of Health’s National Library of Medicine (Craig Locatis and Michael Ackerman) included research in real-time computer vision, computer graphics, and network adaptation strategies, as well as a formal evaluation of the likely effectiveness and acceptance of 3D medical collaboration technology. We have constructed a proof-of-concept prototype system and conducted a between-subject posttest experiment to evaluate the technology’s potential. Development of a production-quality system was beyond the scope of the project. 

## Methods

Three fundamental areas of technology research are required to create a 3DMC system. These are: computer vision methods for reconstruction of a 3D model and/or a user-selectable view of a dynamic scene; remote collaboration display paradigms; and network resource management to support transmission of the 3D view to the remote consultant.

### A. 3D Reconstruction

The 3D reconstruction process involves two major steps: the reconstruction of 3D points from 2D images and the reconstruction of 3D surfaces from the 3D points. To reconstruct 3D points from 2D images we use a novel approach called View-dependent Pixel Coloring (VDPC) [16]. VDPC is a hybrid image-based and geometric approach that estimates the most likely color for every pixel of an image that would be seen from some desired viewpoint, while simultaneously estimating a 3D model of the scene. By taking into account object occlusions, surface geometry and materials and lighting effects, VDPC can produce results where other methods fail: for example, in areas without discernible detail that can be used to derive visual features (texture-less); or in the presence of specular highlights. Both conditions are common in medical scenes. 

We use commercial graphics hardware (PC video cards) to perform the 3D reconstruction very quickly as the video images arrive from the cameras into our computer system. The basic idea is to use the graphics hardware to rapidly project the camera images onto a series of planes swept through geometric space, searching in parallel for the best color matches (least variance between the cameras) at a dense set of points on the planes. The result is a relatively dense point cloud that we can then render in real time, again using the graphics hardware. Additional details about this algorithm can be found in [16, 17].

### B. Remote Collaboration 3D Displays

It is reasonable to expect that in the future medical advisors on duty in a hospital could have access to facilities for stereoscopic, head-tracked viewing of dynamic 3D reconstructions of the remote patient and advisee (as illustrated in Fig. 1a, 1c.). We are working towards this vision. We currently use a simple prototype head-tracked display; it uses a high-resolution monitor and an Origin Instruments opto-electronic system that tracks the viewer’s head position and orientation. The viewer wears a headband with three infrared LEDs that are tracked in real time by a small sensor unit. From this we compute the location of the viewer’s dominant eye and render the reconstructed imagery from that point of view. Thus the viewer can observe the reconstructed scene with natural/intuitive monoscopic head-motion parallax. We are also experimenting with time-multiplexed (shuttered) stereoscopic displays, and with autostereo displays that support multiple simultaneous viewers without glasses.

We want to provide the best possible 3D experience in mobile situations, when the medical advisor is away from the hospital (Fig. 1b & 1d.) For a remote display we are using a personal digital assistant (PDA) because most medical personnel are already accustomed to carrying this type of device. Our goal is to develop or adapt tracking technology and user interface paradigms that will allow a remote medical advisor to use a PDA as a “magic lens” (a paradigm suggested by [18, 19, 20, 21]), providing a view of the remote patient, with natural interactive viewpoint control to help address occlusions and to provide some sense of depth.

**Figure 2. Handheld protoype.**
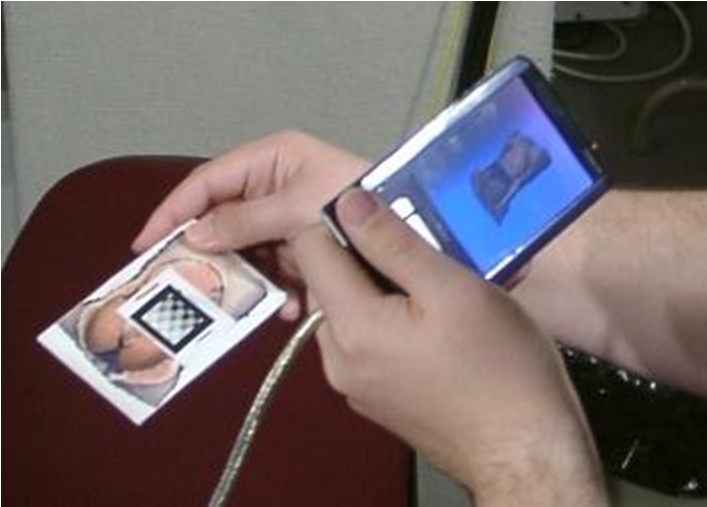
The prototype uses a PointGrey DragonFly [23] camera mounted on the PDA (in the left hand). The prop (in the right hand) has a printed image of our training torso on it, along with a grayscale pattern. We use ARToolkit [24] to track the surrogate with respect to the PDA (remote.)

We are currently investigating a two-handed patient-lens paradigm as shown in Figure 2. Hinckley et al. introduced the idea, using a doll’s head or rubber ball and various tools as ‘props’ for neurosurgeons visualizing patient data [22]. The authors found that users could easily position their hands relative to one another quickly—a task we all do frequently. For 3D medical collaboration, the advisor would have a physical prop that serves as a surrogate for the patient, and a PDA that is tracked relative to the prop. In our prototype, a specially designed PDA cover serves as the prop. The advisor holds the prop (PDA cover) in one hand and the PDA in the other, moving them around with respect to each other as needed to obtain the desired view. This provides the advisor with an instant visual target to aim their “magic lens” at, and also affords new ways of inspecting the remote scene. For example, an advisor can rotate the prop to quickly get a different view, rather than spending time and energy walking around to the other side. As a bonus, tracking a PDA relative to another object is a much more tractable problem than tracking a PDA relative to the world.

We have developed three main software components: a Tracking Server; a PDA Server (that also acts as a client to the Tracking Server); and a PDA Client. The Tracking server receives images from the PDA camera, and uses ARToolKit [24] to track the surrogate (PDA cover) with respect to the PDA. The PDA Server, continually receives a complete representation of the reconstructed patient data from the compute/rendering cluster via a dedicated Ethernet connection as described earlier. The PDA Server also obtains the estimated position and orientation of the PDA from the Tracking Server using the Virtual-Reality Peripheral Network (VRPN) device protocol [25]. It then renders a view of the most recent reconstruction from the estimated PDA position and orientation, and compresses that image such that it can be sent to the PDA. The PDA Client (running on the PDA) receives these compressed images and displays them; it also relays user input back to the PDA Server, such as thumbwheel-controlled field-of-view settings. Each of these components may be run on the same or on separate machines. For additional technical details, see [26].

### C. Network Resource Management

In our target 3DMC scenarios the network path represents a significant bottleneck. We must carefully manage this resource in order to ensure that at all times we transmit the data that are most useful to the overall application and to the goals of the user. In particular, 3DMC has the potential to generate many media streams (camera imagery, tracking data, synthesized views, user interaction and commands, audio, to name just a few) with complex semantic relationships between them. With respect to camera imagery, the utility of the information from one image source may depend on the quality and utility of information from some other source. For example, given two video cameras that share a significant overlap of field of view, it may be preferable to allocate available bandwidth to capture and transmit a high-quality image for only one of the two streams while allowing the quality of the other stream to degrade. Alternatively, it may be better to allocate bandwidth equally in order to achieve similar quality for both streams—useful for VDPC or for other stereo correlation and high-quality 3D reconstruction techniques. Thus the challenge we face is twofold. First, how can we compactly and intuitively specify an adaptation policy to support specific user-level goals? Second, how can we efficiently evaluate that policy? 

We need a framework for addressing the problems of adaptation that is more flexible than previous approaches, which often rely on statically defined priorities (e.g., prioritize audio over video) or simple rule-based decisions (e.g., when available bandwidth is X, do Y). In the framework we are developing, all possible tradeoffs available to the application are mapped as nodes in an N-dimensional “utility space”. Each dimension represents a particular axis for adaptation. Edges between nodes represent both encoding dependencies as well as encoding costs. The nodes and edges form a graph embedded within the utility space. The current information needs of the system are modeled as a “point of interest” within this space. The location of this point of interest changes to reflect how the user is interacting with the system and the dynamics of the application. The utility of any given tradeoff is inversely proportional to the distance between the node that represents the tradeoff and the point of interest. Adaptation is now simply the process by which we select the most useful tradeoff available as defined by the ratio of utility to cost.

The framework is extensible in that new data sources can be modeled within the utility space by simply adding nodes to the graph at the appropriate locations. Similarly, adding new dimensions of adaptation is simply a matter of extending the utility space with another axis and extending the locations of nodes to reflect their position relative to the new dimension. Real-time evaluation is feasible since the adaptation is now a simple mechanical process of maintaining the set of possible tradeoffs in the graph and their distance to the point of interest. Representational dependencies are explicitly modeled by the edges of the graph and constrain our adaptation decisions to those that are feasible given how the data are encoded. Additional technical details can be found in [27].

While the use of a utility space provides us with a mechanical means of driving adaptation and allows parsimonious specification of adaptation policy, the construction of a utility space for a specific application is more art than science. Choices must be made about which adaptation dimensions are going to be modeled, about how these dimensions are scaled relative to each other, about the specific distance function that will be used to establish utility, and about how the actions of the user are reflected by the point of interest. 

For 3DMC, we have identified five dimensions for adaptation: one each for time, resolution, relative change of visual content, and two that capture the notion of region of interest. We are initially limiting ourselves to the problem of adaptation among the different video sources in a 3DMC system capturing the scene. Our current model for each camera assumes that each camera is able to produce low-, medium-, or high-resolution imagery, and that each camera’s view is associated with a 2D field of view within the scene. We are experimenting with simple Euclidean distance functions and greedy allocation algorithms in which the node yielding the maximum utility for the minimum cost is selected until resources are exhausted. The process is iterative and the graph evolves as time passes and new frames are produced. Preliminary experiments show that the system is able to make complex, non-trivial adaptation decisions in an emulated eight-camera setup. Much of the remaining challenge is to develop and evaluate specific utility functions that correspond to the actual perceived quality of real users.

### 3DMC Evaluation

As indicated in the National Academy’s report on telemedicine evaluation [30], it is of critical importance to examine the acceptability and practicality of technology in medicine. Thus we are investigating the potential of 3DMC to improve emergency healthcare and the barriers to its adoption at the present time, early in its lifecycle and before billions of dollars are invested in its implementation.

#### A. Evaluating the potential impact of 3DMC on emergency healthcare

Evaluating the potential of 3DMC technology has unique challenges, some of which can be attributed to the complex context in which emergency situations occur. Patient healthcare priorities, patient privacy, outdoor conditions, and the dynamics of emergency care in the field make it impossible to collect evaluation data in the field. Hence we conducted a controlled experiment evaluation using a posttest between-subjects design. 

 The controlled experiment evaluation compared emergency medical care between three conditions: a paramedic working alone diagnosing and treating a trauma victim; a paramedic and emergency room physician diagnosing and treating a trauma victim collaboratively using state-of-the-art 2D video-conferencing; and a paramedic and emergency room physician diagnosing and treating a trauma victim collaboratively using a 3D proxy. We compared medical outcomes and paramedics’ perceptions between these three conditions. 

**Figure 3:**
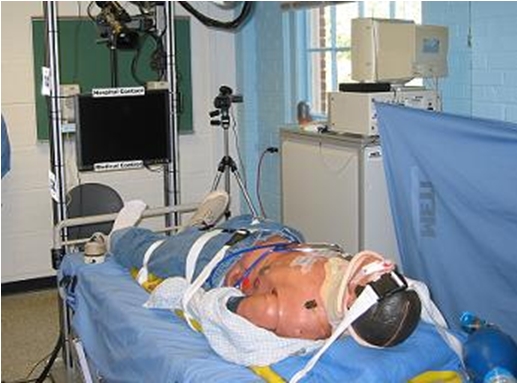
The human patient simulator staged as a car accident victim.

 In each condition, the trauma victim suffered from a difficult airway and a cricothyrotomy was required to save the victim. In a surgical cricothyrotomy an incision is made in the neck, through the skin and the underlying cricothyroid membrane, to allow air to pass to the lungs [31]. Paramedics are trained to manage a difficult airway and perform a surgical cricothyrotomy, yet it is extremely challenging for many paramedics. Even those physicians most experienced in airway management recognize the sense of urgency and anxiety associated with control of the difficult airway, given that trauma victims without an adequate airway will die within minutes if they do not receive appropriate treatment [32]. The inability to secure an airway is the most common cause of preventable death in pre-hospital care [33]. The trauma victim was a METI (http://meti.com/) human patient simulator, or mannequin. This state-of-the-art mannequin can be programmed to act and respond in a life-like manner. For example, its pupils dilate in response to light, its chest rises and falls when breathing, and its heart rate, breathing pattern and blood oxygen levels respond to drug injections and medical procedures. We programmed the mannequin to show all symptoms of the worst-case difficult airway, and we dressed and positioned the mannequin to portray a car accident scenario as realistically as possible as shown in Figure 3 .

The 2D videoconferencing technology used in the experiment was designed in collaboration with emergency room physicians to provide optimal views of the emergency healthcare scenario. Three views of the patient were provided to the collaborating physician using digital cameras directly connected to three 20-inch high-resolution monitors. One camera was a remote-controlled pan-tilt-zoom camera that the advising physician could control. The physicians also had a full-screen view of the patient monitor showing the patient’s heart rate, blood pressure and blood oxygen saturation rates in real time. The physician observed the patient monitor and camera views in a custom-built workstation. In addition, the paramedic had a 2D video view of the consulting physician. 

Because our (indeed all) current 3D reconstruction techniques are relatively limited compared to their expected future potential, we decided to use a 3D proxy condition to assess the 3D paradigm independent of today’s technology—i.e. how effective the 3D reconstruction could be in the future. In the 3D proxy condition, an emergency room physician was physically co-located with a paramedic and victim. The physician had freedom of movement and could point with a laser pointer but was not permitted to touch anything or anyone. Our reasoning for using such a proxy was that if the best possible 3D (reality!) was not medically more effective than 2D video, there would be little reason to continue to pursue the 3D technologies for those purposes.

A total of 60 paramedics, 20 per condition, participated in the experimental evaluation. Each paramedic was first given an introduction to the experiment the mannequin, and the car accident scenario. The paramedic also received a paramedic bag containing medical equipment and available medication. Second, they were asked to approach the trauma car accident victim (i.e., the mannequin) and diagnose and treat him. Each paramedic was free to discuss the diagnosis and treatment as much or as little as they wished with the advising physician. The role of the physician was always played by one of two emergency room physicians. Together, the advising physicians developed a common interaction approach and script, based on their decade-long experience of successful interaction with paramedics, to use when collaborating with the paramedics. A researcher was always in the room with the paramedic, observing the diagnosis and treatment; each session was also videotaped. 

Each session’s videotape was later analyzed to assess medical task performance. The grading protocol used in this analysis was based on standard medical protocols, with details added by two expert emergency room physicians. Each paramedic also completed a questionnaire and participated in a semi-structured interview after their session. In the questionnaire, paramedics reported their perceptions regarding the usefulness of information provided by the physician, the interaction with the physician, and the self-efficacy or the belief in one’s capabilities to perform a task in the future [34]. During the interviews, paramedics reflected on their experiences during their session. 

Details regarding the validity of the experiment design, data collection instruments, data analysis and data analysis results can be found in [35, 36]. A summary of the results is provided in section IV.

#### B. Identifying Barriers to the Adoption and Use of 3DMC 

To identify barriers to the adoption and use of 3DMC within the American healthcare system, we conducted interviews with a variety of actors and stakeholders, including emergency room physicians, residents and nurses in a suburban hospital and a rural medical center, hospital emergency department administrators (responsible for strategic and financial planning, budget, marketing etc.), hospital IT managers and technical support experts, public healthcare (i.e., Medicare) administrators, clinical directors of emergency medical services in suburban and rural areas, an operations manager in charge of county emergency medical services, and a medical director responsible for paramedic training, education, scope of practice and performance improvement on a state level. All of these actors could influence the adoption and use of 3DMC, and/or their jobs could change with its adoption and use. 

At the beginning of each interview, a five minute video that presented our vision for 3DMC was shown. After this introduction, we asked study participants to share their perspective on the benefits and disadvantages of 3DMC for patients, on emergency healthcare professionals, on their department and/or organization, on the healthcare system, and on how the technology might change their current way of working, whether positively and negatively. We used a semi-structured interview protocol that contained open-ended questions, allowing great flexibility and detail to emerge in participants’ responses. A total of 20 interviews were conducted. Three of these interviews were group interviews including 2 or 3 participants, thus a total of 24 people were interviewed. The interviews ranged from 24 to 110 minutes in length, with an average length of 50 minutes. Gender distribution among participants was relatively equal (11 women and 13 men). The interviews were digitally recorded and transcribed. In addition, after each experiment session, we asked paramedics the same questions. We analyzed both sets of interview data to identify challenges facing 3DMC adoption and use.

## Results

### Our 3DMC Prototype System

**Figure 4:**
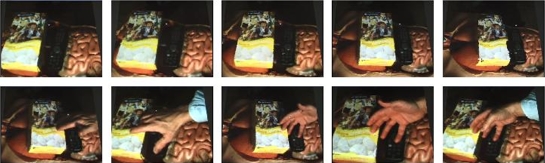
Novel view images reconstructed from camera images. We set a box of Girl Scout cookies on top of the torso to provide more familiar scene geometry. Each image is from a different point in time and from a completely novel viewpoint that does not coincide with any of the cameras used to acquire the raw data.

Figure 4 shows some results of our view-dependent pixel coloring (VDPC) 3D reconstruction. The views were reconstructed online, in real time. Note that the views were reconstructed and rendered from completely novel viewpoints. That is, none of the rendered views matched any of the original camera views at any time. 

**Figure 5:**
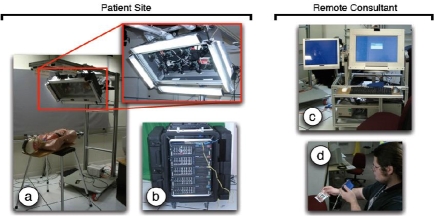
Current 3DMC Prototype System, with patient site components on the left and remote consultant components on the right: (a) camera-lighting array with eight Firewire cameras and high-frequency area lights; (b) compute cluster; (c) a transportable consultant viewing station with 2D and 3D (head-tracked or autostereo) displays; (d) a tracked PDA display.

Figure 5 shows our 3DMC prototype system, which consists of multiple components that would be located at the patient (advisee) site and the remote advisor’s site: (a) a portable camera unit; (b) a portable compute/rendering cluster; and (c, d) two different consultant display devices. 

The portable camera unit (PCU) shown in Fig. 5a is a rolling unit holding a camera-lighting array with eight 640×480 resolution digital (IEEE 1394a) color cameras from Point Grey Research [23]. The cameras are currently mounted in two horizontal rows of four on a portable stand that can be positioned next to a patient. The cameras are positioned so that their visual fields overlap the region of interest on the patient. Mounted around the cameras are multiple high-frequency fluorescent fixtures for flicker-free illumination. The entire array is mounted on a rolling cart with adjustable length and angle, and significant weight (underneath the base) to prevent tipping. The PCU’s power supply includes an AC isolation transformer:^c^ (mounted on the base) to meet the current leakage requirements of UNC Hospital’s medical engineering staff.

The compute/rendering cluster, Fig. 5b, consists of five dual-processor servers in a transportable rack case. Four of the servers are connected to the PCU camera array via Firewire cables. These servers function as Camera Servers, compressing the PCU camera images and forwarding them via a dedicated gigabit Ethernet to the fifth server. Each camera server can optionally record the video streams to disk. The fifth server then decompresses the video streams, loading the color images into texture memory of the graphics card for view-dependent 3D reconstruction as described in Section III.A. Because the PCU and the compute/rendering cluster in our prototype are connected via Firewire cables, they must generally be moved together. In a real production system (in the future), the PCU and compute/rendering servers could be combined into a single unit.

The advisor’s viewing station in Fig. 5c consists of a rolling cart with a dedicated server that is connected to the compute/rendering cluster (4b) by a single gigabit Ethernet cable. This Ethernet cable is the realization of the networking boundary. It is the only link between the compute/rendering cluster (4b) and the consultant viewing station (4c). The connection could be across the hospital or across the world. The station has a high-resolution 2D monitor, an Origin Instruments opto-electronic head tracker, and an autostereoscopic display mounted on an articulated arm. The advisor’s viewing station also includes an AC isolation transformer.

Our current implementation of the tracked PDA mobile display, Fig. 5d, uses a DragonFly camera [23] mounted on a Toshiba e800 PDA. The camera is currently attached to the rendering PC via a Firewire cable, which uses ARToolKit [24] to compute the relative position and orientation of the PDA, as discussed in Section III.B.

The current prototype is not truly portable because of the wired (Firewire) link to a computer, but we could implement the tracking on a PDA with a built in camera in the future. Wagner and Schmalstieg have ported and optimized ARToolKit for PDAs [28, 29], and although their results indicated that the primary bottleneck is image capture rate, new portable devices with cameras better suited to video rate capture are now available. This would allow a wireless interface.

### 3DMC Evaluation

#### A. Evaluating the potential impact of 3DMC on emergency healthcare

The results illustrate that paramedics collaborating with a physician via a 3DMC technology proxy may provide better medical care to trauma victims than paramedics and physicians collaborating via 2D videoconferencing or paramedics working alone.Fewer errors and harmful interventions were performed in the 3D proxy condition. Three paramedics working alone did not perform a cricothyrotomy, although a cricothyrotomy was required to save the trauma victim. A total of eleven harmful interventions were performed when paramedics worked alone, and six were performed when paramedics collaborated with a physician via 2D videoconferencing. In comparison, only two harmful interventions were performed when the collaboration occurred via the 3DMC proxy. 

Although no statistically significant differences with respect to task performance times across conditions emerged from the data analysis, the results from a Levene test for equality of variance indicate that 3DMC technology may overall reduce variation for the total cricothyrotomy performance time. Furthermore, only one (out of five) task performance times in the 3DMC proxy condition was influenced by the number of years of professional experience. In comparison, three and four task performance times were influenced by the length of professional experience when paramedics worked alone or in collaboration via 2D videoconferencing, respectively. Paramedics were assigned to conditions based on their years of professional experience, such that there was an equal distribution of years of professional experience across all three conditions. It appears that the 3DMC technology may reduce differences in diagnosis and treatment caused by differences in years of professional experience, with paramedics with fewer years of experience providing care closer to the level or those with more years of experience. From a trauma victim’s perspective this is an important consideration. In emergency situations patients cannot choose which paramedics will treat them, and patients of course want the most experienced and knowledgeable paramedic to treat them. Similarly, emergency healthcare service organizations want to provide the highest possible level of care, however, there is continual employee turnover with more experienced paramedics retiring and pursuing other career opportunities. The use of 3DMC technology could help reduce the negative impact from lack of experience in providing emergency healthcare.

The statistical results were reflected in comments made by paramedics during post-interviews. Paramedics collaborating with a physician via the 3D proxy expressed unequivocal satisfaction in their cricothyrotomy task performance. In comparison, paramedics working alone or collaborating with a physician via 2D videoconferencing technology tended to express their satisfaction hesitantly or tentatively. 

The results further show that paramedics collaborating with a physician via the 3DMC proxy reported statistically significant higher levels of self-efficacy. Perceptions regarding self-efficacy predict and influence future task performance [34]. Furthermore, the less work experience paramedics in the alone and 2D conditions had, the lower they rated their ability to treat similar patients in the future, whereas work experience had no impact at all on feelings of self-efficacy for paramedics in the 3D proxy condition. This suggests that the 3DMC technology may have a positive impact on future task performance, irrespective of a paramedic’s number of years of professional experience. 

Paramedics also reported that all information provided by the advising physician, except that regarding intubation, was statistically significantly more useful when collaborating in the 3DMC proxy condition than the information provided when collaborating via 2D videoconferencing. In addition to rating the usefulness lower, the paramedics collaborating via 2D videoconferencing showed statistically significantly greater variance in their responses. This variance might be related to previous work experience, in the sense that less experienced paramedics perceived the information from the physician to be more useful than the more experienced ones. However, this correlation was not present in the 3DMC proxy condition. Usefulness of information is an important aspect of emergency medical care because receiving useful information has an impact not only on current task performance, but also on future task performance. 

The paramedics reported that their interaction with the physician was less constrained and better overall when collaborating via the 3DMC proxy condition than when using 2D videoconferencing. Although the interaction when collaborating via the 3DMC proxy was also judged to be good, more accurate and easier, these differences were not statistically significant. 

During interviews, paramedics in both conditions reported an initial awkwardness to their interaction with the physician. However, paramedics in the 2D condition mentioned many more difficulties. As mentioned previously, we followed the advice of expert physicians when positioning the cameras for the 2D videoconferencing. However, we observed that the consulting physician’s view was frequently blocked, e.g., when the paramedic leaned over the victim. This caused the physician to ask unconstructive questions such as: Do you think your incision is big enough? Are you in the airway? No paramedic would purposely make an incision too small or avoid the airway, and this type of questioning appears to hinder physician-paramedic interaction and ultimately patient care. Overall, 75% of the paramedics collaborating using 2D videoconferencing mentioned problems interacting with the physician compared to only 25% of the paramedics collaborating using the 3DMC proxy. Future work includes analysis of the videos from both conditions to gain further insights regarding interaction among the paramedics and physicians.

It appears that the increased depth perception and ability to dynamically change views are important features for 3D telepresence technology. We often saw physicians changing their viewpoint during the experiment sessions, bending down to get a side-angle view, as well as standing up on tiptoe and leaning over the victim. The physicians did not need to ask paramedics to move so they could see the patient better. The paramedic was free to focus on the medical task at hand, and did not need to worry about the physician’s view.

We also saw several features of the 3DMC proxy frequently utilized during the 3D proxy sessions. For example, physicians used the laser pointer to identify the location and size of the required incision, and to point to specific items of medical equipment that the paramedic needed to use. The paramedics paid attention to the physician’s pointing. As Clark discusses [37], the ability to point to physical objects facilitates mutual understanding and task completion. Similarly, the paramedic was free to focus on the medical task at hand, and did not need to worry about the physician’s view. 

However, paramedics also reflected on potential drawbacks the 3DMC technology might introduce. For example, the technology has the potential to make paramedics’ work visible and subsequently evaluated in new ways. Paramedics explained:

It was nice that [the physician] was there and he had your back and he was going to walk you through it. But then again it’s kind of intimidating because you feel like you get trained to do this right…you’re scared you might mess up, and they say, we want you trained better than this.

It kind of makes somebody nervous being monitored by a physician, someone of such higher training. And you’re afraid to make a mistake because this person could be the person that ends up saying [whether] you get to do more, and whether you work or not. 

Ways to avoid these negative consequences that were mentioned by paramedics included opportunities for paramedics and physicians to get to know one another personally and professionally, open and non-judgmental communication practices, and increased understanding regarding joint responsibilities and priorities between paramedics in the field and physicians and nurses in the hospital.

Paramedics’ performance outcomes and perceptions of the technology are only part of the story. Design requirements for a complex technology, such as 3D telepresence technology, that has the potential to affect many professionals and individuals, and which requires substantial changes in our technology and social infrastructures, must take into account as many stakeholders as possible. Meeting the needs of multiple stakeholders will increase the likelihood that the technology will be successfully adopted and used. For example, while technology transparency and ease of use might appear important from a paramedic’s perspective, a hospital administrator might not be willing to invest in a system that does not meet federal and state regulations for protecting patient privacy. An insurance company may not be willing to reimburse for emergency healthcare services utilizing a new technology if the new costs do not provide quantifiable benefits with respect to patient care. To identify these types of requirements, we are conducting interviews with multiple stakeholders in the U.S. healthcare system, including emergency room (ER) physicians and nurses, IT support staff, ER department administrators, and Medicaid administrators. Our goal is to identify multiple stakeholders’ needs and constraints from a broad contextual perspective, in order to provide a more comprehensive set of design requirements than is possible from the data reported in this paper.

Today, paramedics at the scene of an accident collaborate with physicians via radio or cell phone which offer no visual support. Paramedics are required to verbally ‘paint the picture’ of the patient and accident scene to the advising physician in these complex and stressful situations, where the paramedic’s need for information is time-critical and where incorrect decisions based on this information may have fatal consequences. 

Our experimental evaluation illustrates that providing physicians with rich, dynamic visual information of the emergency situation may lead to a more effective collaboration between the physician and paramedic and ultimately to better patient care. Our results illustrate that state-of-the-art 2D videoconferencing does not appear sufficiently flexible to allow a physician to establish and maintain situational awareness of the dynamic and stressful remote emergency healthcare situation effectively. The physician must still ask the paramedic to provide detailed information regarding the patient and the paramedic’s actions, that is, the paramedic must still ‘paint the detailed picture’ for the physician. 

#### B. Identifying Barriers to the Adoption and Use of 3DMC 

Results from the data analysis show that there are both social and technical challenges facing the adoption and use of 3DMC. Challenges were reported by healthcare professionals, healthcare service organizations, and state and federal government healthcare agencies. That is, challenges were foreseen for all segments of the American healthcare system.

Today, the physical boundaries between physicians working in emergency rooms (ERs) and paramedics working in the field and in ambulances mirror the boundaries found in work practices and work cultures between physicians and paramedics. In interviews, paramedics reported that they do not feel respected by physicians. Physicians seem not to listen to them much when they bring a patient into the ER and report the patient’s history and status. They may only call a physician when they are in the field and/or when their medical protocol demands it; otherwise a physician may question their judgment in a derogatory manner. By law, they must do everything that a physician requests, so there is no opportunity for collaboration unless an individual physician chooses to collaborate. By bridging the physical boundaries between paramedics in the field and physicians in the hospital in new ways, 3DMC has the potential to both reduce and increase paramedics’ autonomy and responsibilities. Physicians and the healthcare system could use 3DMC to tightly control paramedics’ medical tasks and career by requiring paramedics to consult with a physician more frequently. Thus, 3DMC that enables paramedics’ work to be more visible both immediately and over time (through digital recordings of 3DMC sessions) could be used to specify tasks paramedics must perform in the field and/or to evaluate and censure their work in a way never before possible. It’s unclear whether such control would benefit patient care over the long term, or whether such control would lead to a reduction of paramedics’ skills and knowledge and consequently to a reduction in the quality of healthcare services provided in the field. A person merely following orders to complete a task may not perform as well as a rather more autonomous person who has some knowledge and skills with respect to the task to be performed. On the other hand, some paramedics believe that if 3DMC made their work in the field more visible, they and the paramedic profession in general would earn more respect and be given increased responsibilities because physicians would gain a better understanding of paramedics’ knowledge and skills. As one paramedic explained:

I think it’s gonna’ benefit us as a profession to have a physician…available…via video. Because they all [say]…we do a great job, but they don't always get what's it really like out there in the field ... having one available to us would give him a better perspective on what we do and it would definitely help our profession... And also ultimately help out the patient…Having some kind of interaction with the physician, and I say physician but it could be nurse, PA or someone that could give the authority to do something…[would] speed up things…You know sometimes we do things in the back of the truck [or ambulance] because the patient needs it right now…But if we had some kind of video conferencing going on, we could possibly do some [additional] things …[to] help in speeding up the process 2 or 3 hours later when they get into the ER… it would have an overall advantage of getting the patient more stable and able to return home, or get better, in a shorter amount of time.

Physicians reported that 3DMC could have a negative impact on their work as well. 3DMC could increase a physician’s workload, and take physicians away from patients already at the ER. Could physicians’ workloads be adjusted to give them sufficient time to collaborate with paramedics in the field? Guidelines and regulations regarding the use of 3DMC that are compatible with paramedics’ and physicians’ work practices and values are required to help ensure that the technology does not have a negative impact on emergency healthcare services.

A 3DMC technical requirement identified by paramedics and physicians alike during interviews is near-instantaneous set up and start times for the technology. In emergencies, the quicker care is provided to patients, the quicker patients recover. This can reduce patient suffering, lower costs and enable patients to return to work more quickly. Thus paramedics and physicians do not want to spend time setting up and/or booting 3DMC equipment. Turning it on by a single switch would be ideal. Fulfilling this requirement may mean that the equipment could be mounted in ambulances. Situating the technology within an ambulance would also greatly reduce or eliminate audio interference caused by noise from other vehicles, from bystanders and from weather conditions in the field. Of course, when an ambulance is in motion and the technology is in use, vibrations could cause visual problems for physicians. Advanced shock-resistant mountings and image stabilization techniques may be needed. 

A portable version of the technology would also be useful in situations when the patient cannot be moved into an ambulance. Portable versions (cf. Fig. 1, left column, center) would need to be protected from the weather (rain, snow, winds), be impact resistant (in case the system is dropped), and perhaps be battery-operated. Some study participants suggested that having different levels of visual resolution, clarity and color accuracy available for different situations could be feasible. For example, when a portable unit or a unit in a poorly-networked geographical region is being used, lower levels of resolution and lower transmission speeds may be acceptable. When no other options are available, physicians and paramedics could still benefit from 3DMC under these circumstances.

For organizations, 3DMC raises issues concerning legal responsibility and liability. If patient care becomes a collaboration between a paramedic and a physician, who is legally responsible for patient outcomes? Would the physician who has more training and skills be held responsible even though the physician could not physically touch and treat the patient? Or would the paramedic following (or misunderstanding or ignoring) the physician’s advice be held responsible? Could 3DMC sessions legally occur across state borders, or would they be limited by state boundaries? This is an issue facing many new medical technologies that span distances, such as remote surgery. Furthermore, would the 3DMC session become part of a patient’s record? Each session could be recorded and archived for future access. In certain situations, patient care could be improved if an attending physician could see the patient’s earlier condition and observe the treatment performed in the field (or ambulance). However, would patients and lawyers use the recorded sessions as a weapon to sue paramedics and physicians, increasing the number and complexity of malpractice lawsuits? On the other hand, could physicians and paramedics use recorded sessions to more successfully defend their decisions and actions? Integrity of, and long term access to, recorded sessions may emerge as future challenges.

Another challenge for organizations concerns the cost of purchasing and operating 3DMC technology. Emergency medical services (EMS) providers typically have smaller operating budgets than medical centers, yet they would need to buy camera and transmission equipment for each EMS vehicle or ambulance. In contrast, a larger medical center may only have to buy equipment to support one or two viewing stations or viewing devices. As mentioned above, future viewing devices may even be small handheld devices, with relatively low costs.

Telecommunications network costs also are a concern. Who will pay for development, implementation and operational costs for the advanced telecommunications networks needed to support 3DMC? As discussed above, the network will need new capabilities to be able to transmit the visual and audio information. It should also be robust, to ensure 24/7 availability, and secure, to protect patient privacy. Such networks may evolve in large populated areas because other business, research and/or government needs will justify their costs, as has happened historically. But 3DMC may make most sense medically if coverage can be provided to rural areas, from where transport time to large medical centers with specialized expertise is long. Paramedics reported that today, in parts of North Carolina, they have neither radio nor cell phone coverage. This issue is not one that can be easily resolved by individual EMS providers or by large medical centers alone. Can local, state and federal government agencies, businesses and research work together to decrease the digital divide that exists today, and enable new technologies and services such as 3DMC to reach all in need?

In the U.S., billing for 3DMC services would present a unique challenge. Insurance companies, as well as state and federal healthcare agencies, such as Medicare, that are billed for patients’ medical expenses, need rigorous evidence that 3DMC improves patient healthcare and is cost-effective, i.e., does not increase medical costs, before they will agree to pay for 3DMC services. Study participants reported that expensive clinical patient trials running for long periods of time would be required. Funding such trials will be challenging. Infrastructure changes that are not typically part of clinical drug trials may be required to this end. That is, a clinical trial could require changes to local telecommunication networks, to hospital and EMS information technology infrastructure, to paramedic and physician training, to emergency treatment protocols, and to procedures for obtaining patient consent in emergency situations. 

But it is not all bad news. In addition to the uses and benefits for 3DMC that we, as researchers, initially envisioned, study participants identified further uses and benefits. As mentioned above, 3DMC could be helpful in assisting healthcare services in developing countries. It could also be used to enhance collaboration between physicians at large and smaller medical centers. As a physician explained:

I take between 1 and 10 calls in an 8-hour period…from other physicians trying to transfer patients. And to be able to utilize [3DMC] technology to…see that patient, and talk about …what we can offer that patient would be useful…I see this as a bigger application...than pre-hospital [care].

When physicians have an increased understanding of patients’ needs, they can better determine whether a patient could be treated in their local community, without transfer to a large medical center. This could save costs. It could allow patients to receive better emotional healing support. When patients are kept in their community, they are able to have more frequent contact with their loved ones. When a patient must be transferred to a large medical center and physicians have an in-depth understanding of the patient’s needs, resources can immediately be scheduled for that patient. This could eliminate delays in treatment, decrease the length of hospital stays and help make better use of hospital resources in general. 

An additional, indirect benefit from collaboration via 3DMC could be educational. Study participants reported that both physicians at large and small medical centers and paramedics could learn from each other during 3DMC sessions. Paramedics and physicians at smaller medical centers could learn new skills from physicians at larger medical centers who have better access to new medical techniques and perhaps more experience. Physicians at large medical centers could learn more about emergency care situations in the field, increasing their ability to diagnose and treat patients when they arrive at the ER. Recorded sessions, with anonymity of participants preserved, could also be used as rich case studies in medical training courses.

Several participants also suggested that 3DMC could be used as a marketing tool and status symbol to attract patients and their families. For example, retirees who typically use more healthcare services than other groups may be persuaded to move to a rural area that uses 3DMC. Because large medical centers are typically located in populated areas with a high cost of living, a rural area with a lower cost of living and 3DMC that enables high quality medical care could be attractive to retirees. In urban areas where several hospital and EMS providers compete for patients, 3DMC may help differentiate between healthcare providers. Patients with higher incomes and good insurance coverage may choose to use EMS providers and hospitals that provide the high quality emergency healthcare that 3DMC (ideally) enables.

In summary, there are many benefits and challenges with respect to 3DMC. Government mandates and/or strong financial incentives provided by the government and other interest groups could well outweigh the challenges identified by the study participants. In countries that have socialized medical systems and government-owned or -regulated telecommunications companies, these types of challenges may be easier to overcome. 

## Conclusions

In the 2001 PITAC report to the President, Transforming Health Care Through Information Technology, recommendation 5 states that the Department of Health and Human Services should establish an “aggressive research program in computer science” that addresses “long-term needs, rather than the application of existing information technology to biomedical problems” [38, p. 13]. 3DMC is an aggressive research program addressing long-term needs for more effective medical collaboration, improved healthcare, and (ideally) reduced medical care costs. It is interdisciplinary, uniquely bringing together researchers in computer science, information science and medicine. This paper synthesizes the progress made towards developing and evaluating 3DMC. 

We have made progress developing computer vision methods for reconstruction of a 3D model/view of a dynamic scene, remote collaboration displays, and network resource management algorithms to support transmission of 3D views. The results were realized in a prototype system, demonstrating that with a future, larger-scale technological effort, the technology could be brought within our reach and embedded within our medical infrastructure. 

We have made progress in understanding the potential of 3DMC. We did not find statistically significant evidence that medical task performance improved with 3DMC when compared to 2D videoconferencing, yet there is statistically significant evidence that paramedics preferred 3DMC over 2D videoconferencing. There is also evidence that self-efficacy is significantly lower after a paramedic collaborated with a physician using 2D videoconferencing. These are important results as perceptions of new technology and self-efficacy are strong indicators of adoption of technology and future task performance.

We have also identified additional benefits to and challenges facing 3DMC. Typically such challenges are discovered after considerable resources are spent developing and implementing new technology, and people’s lives are negatively impacted. Identifying challenges earlier enables the technology design to address the challenges at a lower cost; changing technology after it has been designed and developed increases its cost. It also reduces the likelihood of harmful unintended consequences.

The Office for the Advancement of Telehealth [38] points out how the Telecommunications Act of 1996 has and continues to improve access and reduce costs for urban and rural healthcare providers. Our project builds on this tradition, discovering new ways technology can be used to provide emergency healthcare outside hospital settings to trauma victims. Trauma is a significant health problem, frequently referred to as the ‘hidden epidemic of modern society’ because it is responsible for more productive years lost than heart disease, cancer and stroke combined [39, 40]. 3DMC can potentially bring needed healthcare expertise to trauma victims before they are transported to hospitals. The sooner a victim receives appropriate expert medical care, the shorter their recovery time and lower their medical care costs. Thus 3DMC could have a significant impact on patient healthcare in the future. Additional details can be found in discipline-specific papers [see 16, 17, 26, 27, 35, 36].

## Footnotes

a Binocular disparity is the difference between a matching pair of image feature locations as seen by the left and right eyes. The differences are one of the cues that the brain uses to estimate the distance to the feature, i.e. the distance to and shape of objects.

b Motion parallax is the apparent dynamic visual displacement of an object as one translates their head or the object.

c An isolation transformer is a special AC power unit that contains components to prevent electrical current from flowing from one side of the unit to the other. This isolation provides an added level of electrical safety.

## Appendix

### Appendix 1 List of Abbreviations Used

2D Two‐dimensional

3D Three‐dimensional

3DMC 3D medical consultation

AC Alternating current

ER Emergency room

EMS Emergency medical services

GRA Graduate Research Assistant

LED Light emitting diode

PC Personal computer

PCU Portable camera unit

PDA Personal digital assistant, typically a handheld device

PITAC President's Information Technology Advisory Committee

VDPC View‐Dependent Pixel Coloring

VE Virtual environment

VRPN Virtual‐Reality Peripheral Network

## References

[1] Chu Y, Huang X. and Ganz A. (2005). WISTA: A wireless transmission system for disaster patient care remote 3D medical consultation. Proc. of BROADNETS: 2nd IEEE/CreateNet International Conference on Broadband Networks.

[2] Stokes D E (1997). Pasteur’s Quadrant.

[3] Permanente Kaiser (2000). Outcomes of the Kaiser Permanente tele-home health research project. Archives of Family Medicine.

[4] Dimmick S L, Mustaleski C, Burgiss S G, Welsh T S (2000). A case study of benefits & potential savings in rural home telemedicine. Home Healthc Nurse.

[5] Doty E, Zincone J L H, Balch D C, Weghorst Eds J, Sieburg Hans B, Morgan Karen S (1996). Telemedicine in the North Carolina prison system. In Medicine Meets Virtual Reality: Healthcare in the Information Age. IOS.

[6] Ellis D G, Mayrose J, Jehle D V, Moscati R M, Pierluisi G J (2001). A telemedicine model for emergency care in a short-term correctional facility. Telemedicine Journal and e-Health.

[7] Kienzle M G (2000). Rural-academic integration: Iowa’s national laboratory for the study of rural telemedicine. National Laboratory for the Study of Rural Telemedicine, Tech. Rep.

[8] Agha Z, Schapira R M, Maker A H (2002). Cost effectiveness of telemedicine for the delivery of outpatient pulmonary care to a rural population. Telemed J E Health.

[9] Walt Disney Pictures ; directors, Pixote Hunt ... [et al.] ; producer, Donald W. Ernst ; host sequences written by Don Hahn, Irene Mecchi, David Reynolds (2000). Project Phoenix: Scrutinizing a telemedicine testbed. Final Project Report. National Library of Medicine, Contract.

[10] Tachakra S (2001). Depth perception in telemedical consultations. Telemed J E Health.

[11] Hendrix C, Barfield W (1996). Presence within virtual environments as a function of visual display parameters.

[12] Snow MP (1996). Charting presence in virtual environments and its effects on performance.

[13] Pausch R, Shackelford M A, Proffitt D (1993). A user study comparing head-mounted and stationary displays. Proc. of IEEE Symposium on Research Frontiers in Virtual Reality.

[14] Schroeder R, Steed A, Axelsson AS, Heldal I, Abelin A, Wideström J, Nilsson A, Slater M (2001). Collaborating in networked immersive spaces: As good as being there together?. Computers & Graphics, Special Issue on Mixed Realities - Beyond Conventions.

[15] Mizell W D, Jones P S, Slater M, Spanlang B (2002). Comparing immersive virtual reality with other display modes for visualizing complex 3D geometry.

[16] Yang R (2003). View-dependent pixel coloring—a physically-based approach for 2d view synthesis.

[17] Yang R, Pollefeys M, Yang H, Welch G (2004). A unified approach to real-time, multi-resolution, multi-baseline 2d view synthesis and 3d depth estimation using commodity graphics hardware. International Journal of Image and Graphics (IJIG).

[18] Fitzmaurice W G (1993). Situated information spaces and spatially aware palmtop computers. Communications of the ACM.

[19] Fitzmaurice W G, Buxton W (1994). The chameleon: Spatially aware palmtop computers. Proc. ACM CHI.

[20] Mohring M, Lessig C, Bimber O (2004). Video see-through AR on consumer cell-phones. Proc. 3rd IEEE and ACM International Symposium on Mixed and Augmented Reality (ISMAR’04).

[21] Pasman W, van der Schaaf A, Lagendijk R L, Jansen F W (1999). Accurate overlaying for mobile augmented reality. Computers and Graphics.

[22] Hinckley K, Pausch R, Goble J C, Kassell N F (1994). Passive real-world interface props for neurosurgical visualization. Proc. ACM CHI.

[23] Research Point Grey. http://www.ptgrey.com.

[24] ARToolKit. http://www.hitl.washington.edu/artoolkit.

[25] Virtual reality peripheral network.

[26] Welch G, Noland M, Bishop G (2007). Complementary tracking and two-handed interaction for remote 3D medical consultation with a PDA. Proceedings of Trends and Issues in Tracking for Virtual Environments, Workshop at the IEEE Virtual Reality 2007 Conference.

[27] Gotz D, Mayer-Patel K (2004). A general framework for multidimensional adaptation. MULTIMEDIA ’04: Proc. 12th Annual ACM international conference on Multimedia.

[28] Wagner D, Schmalstieg D (2003). First steps towards handheld augmented reality. Proc. 7th IEEE International Symposium on Wearable Computers International Symposium on Wearable Computers.

[29] Wagner D, Schmalstieg D, Welch G (2006). Handheld augmented reality displays. Proc. 2nd Emerging Display Technologies Workshop (EDT 2006).

[30] Field M (1996). Telemedicine: A guide to assessing telecommunications for health care.

[31] American Society of Anesthesiologists Task Force on Difficult Airway Management (2003). Practice guidelines for management of the difficult airway: an updated report by the American Society of Anesthesiologists Task Force on Management of the Difficult Airway. Anesthesiology.

[32] Gawande A (2001). Complications.

[33] Bair A E, Panacek E A, Wisner D H, Bales R, Sakes J C (2003). Cricothyrotomy. J Emerg Med.

[34] Bandura A (1997). Self-efficacy: the exercise of control.

[35] Sonnenwald D H, Söderholm H M, Cairns B, Manning J E, Welch G, Fuchs H (2009). Exploring the potential of video technologies for collaboration in emergency medical care: Part I. Information sharing. Journal of the American Society of Information Science & Technology.

[36] Söderholm H M, Sonnenwald D H, Cairns B, Manning J E, Welch G, Fuchs H (2009). Exploring the potential of video technologies for collaboration in emergency medical care: Part II. Task performance. Journal of the American Society of Information Science & Technology.

[37] Clark H (1996). Using Language.

[38] Office for the Advancement of Telehealth (2001). Telemedicine report to congress. U.S. Department of Health and Human Services, Health Resources and Services Administration, Tech. Rep.

[39] Meyer A (1998). Death and disability from injury: a global challenge. J Trauma.

[40] Coates T J, Goode A (2001). Towards improving prehospital trauma care. Lancet.

[41] Welch G, Sonnenwald D, Mayer-Patel K, Yang R, State A, Towles H, Cairns M B, Fuchs H (2005). Remote 3D medical consultation. Proceedings of BROADNETS: 2nd IEEE/CreateNet International Conference on Broadband Networks.

